# Maternal Ramadan fasting and fetal cardiac function: subclinical hemodynamic alterations revealed by doppler evaluation

**DOI:** 10.1186/s12884-026-08683-4

**Published:** 2026-02-05

**Authors:** Deniz Taşdemir

**Affiliations:** Department of Perinatalogy, Şanlıurfa Training and Research Hospital, Yenice Neighborhood, Yenice Road No:1, Şanlıurfa, 63250 Turkey

**Keywords:** Ramadan fasting, Pregnancy, Fetal cardiac function, Myocardial performance index (MPI), Doppler ultrasonography, Amniotic fluid index (AFI)

## Abstract

**Background and objectives:**

To assess the association between maternal Ramadan fasting and fetal cardiac function and hemodynamics using comprehensive Doppler echocardiography, with a focus on subclinical myocardial and circulatory changes.

**Materials and methods:**

In this cross-sectional Doppler ultrasound study, 203 healthy singleton pregnancies between 24 and 32 weeks of gestation were examined, comprising 102 women who fasted for ≥ 10 days during Ramadan and 101 non-fasting controls. The study was prospectively registered with the National Clinical Trial **(**ClinicalTrials.gov Identifier: NCT06900257, registration date 23 March 2025). Doppler assessments included umbilical artery, middle cerebral artery, and ductus venosus pulsatility indices (PI), cerebroplacental ratio, and fetal cardiac parameters, including left and right myocardial performance indices (LV MPI, RV MPI), tricuspid and mitral annular plane systolic excursions (TAPSE, MAPSE), cardiothoracic ratio (CTR), and amniotic fluid index (AFI). Statistical analyses were performed using IBM SPSS v25.0 and Python 3.10, including group comparisons, correlation analysis, multivariable regression, and receiver operating characteristic (ROC) analysis.

**Results:**

Fasting pregnancies demonstrated significantly higher MCA PI (*p* < 0.001), LV MPI (*p* < 0.001), RV MPI (*p* = 0.041), and CTR (*p* < 0.001), and lower AFI (*p* < 0.001) than controls. Umbilical and ductus venosus PI, TAPSE, and MAPSE did not differ significantly.LV MPI correlated positively with CTR (r = 0.33) and inversely with AFI (r = –0.42). In multivariable regression analysis, fasting status was independently associated with higher LV MPI and lower AFI. ROC analysis demonstrated that LV MPI had no clinically meaningful predictive value for low amniotic fluid volume (AUC = 0.52).

**Conclusions:**

Maternal Ramadan fasting was associated with mild differences in fetal cardiac and hemodynamic parameters, characterized by increased MPI and MCA PI and reduced AFI, without overt Doppler evidence of fetal distress. These findings highlight the adaptive capacity of the fetal cardiovascular system; however, given the cross-sectional design, causality and reversibility cannot be established, but support individualized monitoring during fasting in pregnancy.

**Trial registration:**

ClinicalTrials.gov Identifier NCT06900257. Registered on 23 March 2025. (The record currently appears as “Update Not Released” on ClinicalTrials.gov due to the temporary U.S. government shutdown, but the submission has been completed and will be publicly released once the database reopens.)

**Supplementary Information:**

The online version contains supplementary material available at 10.1186/s12884-026-08683-4.

## Introduction

Ramadan fasting, observed by millions of Muslims worldwide, entails abstaining from food and fluids from dawn to sunset. Although pregnant women are religiously exempt, many continue fasting for cultural and personal reasons [1–3]. Pregnancy, however, is a state of increased metabolic demand and fluid requirement, making prolonged fasting a potential physiological stressor that may be associated with changes in fetal well-being [4]. 

Previous studies have examined the effects of Ramadan fasting on maternal metabolism, fetal growth, and amniotic fluid volume, with largely inconsistent results [5–7]. While most report no adverse perinatal outcomes, subtle metabolic shifts such as reduced glucose availability and maternal hydration have been hypothesized to influence fetal circulation [8]. 

Fetal cardiac performance can be objectively assessed by Doppler-derived indices including the myocardial performance index (MPI), tricuspid and mitral annular plane systolicexcursions (TAPSE, MAPSE), and cerebral–umbilical resistance ratios [9–11]. These parameters are considered sensitive markers of subclinical hemodynamic differences, even in the absence of overt fetal distress. However, data comparing these parameters between fasting and non-fasting pregnancies during Ramadan remain limited. 

Therefore, this study aimed to compare fetal cardiac function and hemodynamicparameters between fasting and non-fasting pregnancies during Ramadan using detailed. Doppler assessment. Given the cross-sectional design, the study focuses on associations. rather than fetal cardiovascular responses or causal effects.

## Materials and methods

This study was conducted between January 2025 and July 2025 at the Perinatology Outpatient Clinic of Şanlıurfa Training and Research Hospital, following approval by the Institutional Ethics Committee (Approval No: HRÜ/25.03.01]).

This cross-sectional observational study was registered with the National Clinical Trial (ClinicalTrials.gov Identifier: NCT06900257, registration date 23 March 2025) prior to participant enrollment to ensure transparency and adherence to international research standards.

All participants provided written informed consent prior to enrollment, and the study was conducted in accordance with the Declaration of Helsinki.

### Study population

A total of 203 healthy pregnant women between 24 and 32 weeks of gestation were enrolled.

and stratified into two groups:

- Fasting group (n = 102): women who observed Ramadan fasting for ≥10 consecutive days, verified by self-report and fasting logs.asdas
- Control group (n = 101): women who did not fast during the same period and werematched for maternal age (±2 years), gravidity, and gestational week (±1 week).asd

Inclusion criteria were: singleton pregnancy, absence of structural or chromosomal fetal anomalies, normal first- and second-trimester screening, and regular antenatal follow-up.

Exclusion criteria included pre-existing diabetes mellitus, hypertension, thyroid or renal disease, polyhydramnios/oligohydramnios, intrauterine growth restriction, smoking, and use of medications affecting cardiovascular or metabolic status.

Maternal demographic and obstetric data were recorded, including age, parity, pre-pregnancy BMI, gestational week, and number of fasting days.

### Ultrasound and doppler protocol

All sonographic assessments were performed by a single maternal–fetal medicine specialist with > 10 years of experience, using a GE Voluson E8 Expert^®^ system equipped with a 2–9 MHz convex transducer (C2-9-D).

To minimize metabolic variability, examinations in the fasting group were scheduled within the last two hours of the fasting period (typically between 5:00–7:00 PM), and examinations in the control group were performed during the same time window.To reduce observer bias, the operator was blinded to the participants’ fasting statusduring amniotic fluid and Doppler measurements. 

Fetal biometric measurements (BPD, FL, AC, EFW) were recorded, followed by Doppler evaluations performed under controlled conditions:

- Insonation angle: <30°, optimized for each vessel
- Fetal activity: absent or minimal movement
- Sweep speed: 5 cm/s
- Wall motion filter: 300 Hz
- Sample volume: 3–4 mm

For each vessel, at least six consecutive uniform waveforms were obtained and averaged.

### Vascular doppler parameters

1. Umbilical artery (UA): sampled from a free-floating cord segment.
2. Middle cerebral artery (MCA): measured at the proximal third near its origin from the circle of Willis.
3. Ductus venosus (DV): visualized in the midsagittal plane, confirming triphasic waveform morphology.

The pulsatility index (PI) was calculated for each vessel.

The MCA/UA PI ratio served as an index of cerebroplacental resistance, where lower values suggest fetal circulatory redistribution.

### Cardiac functional assessment

Fetal cardiac function was analyzed using both spectral Doppler and M-mode echocardiography:

- Mitral and tricuspid inflow (E and A waves) measured from apical four-chamber view to calculate E/A ratios.
- The fetal myocardial performance index (MPI) was measured using a modified Doppler technique with simultaneous acquisition of ventricular inflow and outflow waveforms within the same cardiac cycle.
- The sample volume was positioned to include both the atrioventricular valve inflow and the ventricular outflow tract, allowing synchronized measurement of IVCT, IVRT, and ET. A representative example of the MPI measurement using simultaneous inflow–outflow Doppler acquisition is provided in Supplementary Figure S1.
- The fetal MPI was calculated as (IVCT + IVRT) / ET, where IVCT is the isovolumetric contraction time, IVRT the isovolumetric relaxation time, and ET the ejection time [11].
- Individual IVCT, IVRT, and ET durations were recorded and analyzed separately for intergroup comparison.
- TAPSE and MAPSE were obtained using M-mode echocardiography, with the sampling cursor carefully aligned parallel to the direction of atrioventricular annular motion at the lateral tricuspid and mitral annulus, respectively.
- All TAPSE and MAPSE values were verified and recorded in millimeters (mm).
- CTR and Ventricular Sphericity Indices (LVSI, RVSI) were calculated to assess cardiac geometry and remodeling.
- Amniotic Fluid Index (AFI) was determined by the four-quadrant method and recorded in centimeters.

All measurements were averaged from three consecutive cardiac cycles to minimize intraobserver variability.

Intraobserver variability was assessed in a random subset of measurements and was maintained below 5%.

### Statistical analysis

All data were analyzed using IBM SPSS Statistics version 25.0 (IBM Corp., Armonk, NY, USA) and cross-validated with Python 3.10 (Pandas, Statsmodels, SciPy) for advanced statistical analyses.Continuous variables were expressed as mean ± SD and 95% confidence intervals (CI).

The Kolmogorov–Smirnov test was used to assess data normality. 

For group comparisons:

- Independent Samples t-test was applied for normally distributed data. including the individual components of MPI (IVCT, IVRT, and ET).
- Mann–Whitney U test for non-parametric data.
  Categorical variables were analyzed with the Chi-square test.
  Statistical significance was set at p < 0.05.

To strengthen analytical robustness beyond mean comparison, multiple complementary analyses were incorporated:

- Effect Size (Cohen’s *d*): 
  Quantified the magnitude of intergroup differences for major parameters (MCA PI, LV MPI, RV MPI, CTR, AFI). 
  Effect sizes were interpreted as small (0.2), medium (0.5), or large (≥ 0.8).
- Correlation Analysis: 
  Spearman’s rank correlation (rₛ) was employed to explore relationships among fetal cardiac and hemodynamic parameters (LV MPI, RV MPI, CTR, AFI, MCA PI).
  Correlations were categorized as weak (r < 0.3), moderate (0.3–0.5), or strong (>0.5).
- Multiple Linear Regression:
  Separate regression models were built for LV MPI and AFI as dependent variables. Independent predictors included fasting status (binary), gestational age (weeks), and fetal heart rate. 
  Model adequacy was tested using R², adjusted R², β coefficients, and 95% CI. Multicollinearity was excluded with variance inflation factor (VIF < 2.0).
- Receiver Operating Characteristic (ROC) Analysis: 
  The discriminative capacity of LV MPI in identifying low amniotic fluid (AFI < 10 cm) was assessed.
  The area under the curve (AUC) was calculated with 95% CI; values of 0.5 indicate no discrimination, 0.7–0.8 acceptable, and >0.8 excellent. 
  The optimal cut-off was derived using Youden’s Index (J = Sensitivity + Specificity – 1).
- Power and Sensitivity Analysis: 
  Post hoc power calculations demonstrated an achieved statistical power of >0.85 for significant comparisons (LV MPI, CTR, AFI), confirming adequate sample size.

## Results

A total of 203 singleton pregnancies were included in the final analysis, comprising 102 fasting and 101 control participants. The mean gestational age at assessment was similar between groups (27.1 ± 2.6 vs. 26.3 ± 2.1 weeks, *p* = 0.056), whereas the mean fasting duration among fasting women was 21.7 ± 4.0 days (Table 1).

**Table 1:** Comparison of gestational age and fasting duration between fasting and control groups

Parameter	Fasting Group (*n* = 102)	Control Group (*n* = 101)	*p* value
Gestational age (weeks)	27.1 ± 2.6 (24–34) [95% CI: 26.6–27.7]	26.3 ± 2.1 (24–31.5) [95% CI: 25.9–26.8]	0.056^a^
Fasting duration (days)	21.7 ± 4.0 (13–29) [95% CI: 20.9–22.5]	—	—

Continuous variables are presented as mean ± standard deviation (range) and 95% confidence interval (CI)

*Abbreviations*: *CI* Confidence Interval

^a^ Mann–Whitney U test was used for non-normally distributed data

### Fetal doppler hemodynamics

Umbilical artery pulsatility index did not differ significantly between fasting and control groups. Ductus venosus pulsatility index was also not significantly different; a borderline trend toward higher values was observed in the fasting group (*p* = 0.054). In contrast, the middle cerebral artery PI was significantly higher in fasting pregnancies (2.13 ± 0.17 vs. 2.05 ± 0.14, *p* < 0.001), indicating increased cerebral vascular resistance. Similarly, the MCA/UA PI ratio was significantly elevated in the fasting group (2.12 ± 0.35 vs. 2.01 ± 0.25, *p* = 0.010) (Table 2).

**Table 2:** Comparison of fetal doppler pulsatility index (PI) parameters between fasting and control groups

Parameter	Fasting Group (*n* = 102)	Control Group (*n* = 101)	*p* value
Umbilical artery PI	1.02 ± 0.16 (0.65–1.36) [95% CI: 0.99–1.06]	1.03 ± 0.15 (0.74–1.35) [95% CI: 1.03–1.06]	0.890^a^
Middle cerebral artery (MCA) PI	2.13 ± 0.17 (1.82–2.64) [95% CI: 2.10–2.17]	2.05 ± 0.14 (1.79–2.45) [95% CI: 2.02–2.07]	< 0.001^b^
Ductus venosus (DV) PI	0.77 ± 0.15 (0.41–1.34) [95% CI: 0.74–0.80]	0.73 ± 0.10 (0.54–1.05) [95% CI: 0.71–0.75]	0.054^b^
MCA PI / Umbilical PI ratio	2.12 ± 0.35 (1.44–3.26) [95% CI: 2.05–2.19]	2.01 ± 0.25 (1.44–2.64) [95% CI: 1.96–2.06]	0.010^b^

Data are expressed as mean ± standard deviation (range) and 95% confidence interval (CI)

*Abbreviations*: *PI* Pulsatility Index, *MCA* Middle Cerebral Artery, *DV* Ductus Venosus, *CI* Confidence Interval

^a^ Mann–Whitney U test; ^b^ Student’s *t*-test

### Fetal cardiac function and morphology

No significant differences were found in mitral or tricuspid E/A ratios, TAPSE, or MAPSE values between groups (*p* > 0.05). In contrast, both left and right ventricular myocardial performance indices (LV MPI and RV MPI) were significantly higher among fasting women (0.54 ± 0.08 vs. 0.49 ± 0.08, *p* < 0.001; 0.56 ± 0.09 vs. 0.53 ± 0.10, *p* = 0.041, respectively), suggesting mild subclinical cardiac loading. Cardiothoracic ratio (CTR) was notably increased in the fasting group (0.29 ± 0.02 vs. 0.23 ± 0.04, *p* < 0.001), while amniotic fluid index (AFI) was markedly lower (11.24 ± 1.41 vs. 17.92 ± 1.60 cm, *p* < 0.001) (Table 3). To further delineate the components contributing to the observed increase in left myocardial performance index, individual isovolumetric time intervals were analyzed. Fasting pregnancies demonstrated a significant prolongation of isovolumetric relaxation time (IVRT) compared with controls (54.2 ± 5.2 vs. 48.6 ± 4.5 ms, *p* < 0.001), whereas no significant difference was observed in isovolumetric contraction time (IVCT) (36.4 ± 3.9 vs. 36.1 ± 3.8 ms, *p* = 0.584). Ejection time (ET) was modestly but significantly shorter in the fasting group (167.8 ± 11.5 vs. 172.8 ± 10.9 ms, *p* = 0.002), indicating that the higher MPI values were primarily driven by altered diastolic relaxation rather than impaired systolic contraction. In contrast, right ventricular isovolumetric contraction time (IVCT), isovolumetric relaxation time (IVRT), and ejection time (ET) did not differ significantly between fasting and control groups (all *p* > 0.05), suggesting that the modest increase in RV MPI was not attributable to a dominant alteration in a single time interval (Table 3).

**Table 3:** Comparison of fetal cardiac function and morphological parameters between fasting and control groups

Parameter	Fasting Group (*n* = 102)	Control Group (*n* = 101)	*p* value
Mitral E (cm/s)	32.78 ± 7.37 (17.9–46.4)	31.87 ± 4.59 (20.1–43.2)	0.294^b^
Mitral A (cm/s)	50.67 ± 8.61 (30.4–67.4)	49.87 ± 7.09 (32.6–69.7)	0.471^b^
Mitral E/A ratio	0.64 ± 0.07 (0.47–0.79)	0.64 ± 0.05 (0.51–0.77)	0.831^a^
Left ventricular MPI	0.54 ± 0.08 (0.30–0.74)	0.49 ± 0.08 (0.31–0.73)	< 0.001^b^
LV IVCT (ms)	36.4 ± 3.9 (28.7–44.8)	36.1 ± 3.8 (29.4–45.2)	0.584^b^
LV IVRT (ms)	54.2 ± 5.2 (41.6–67.9)	48.6 ± 4.5 (39.8–60.7)	< 0.001^b^
LV ET (ms)	167.8 ± 11.5 (143.2–196.4)	172.8 ± 10.9 (149.6–198.1)	0.002^b^
Tricuspid E (cm/s)	33.41 ± 5.86 (20.5–47.1)	34.45 ± 5.56 (23.1–59.3)	0.195^b^
Tricuspid A (cm/s)	50.62 ± 7.10 (35.2–64.6)	52.55 ± 7.10 (33.3–76.6)	0.055^b^
Tricuspid E/A ratio	0.65 ± 0.07 (0.50–0.82)	0.65 ± 0.05 (0.50–0.85)	0.485^a^
Right ventricular MPI	0.56 ± 0.09 (0.36–0.75)	0.53 ± 0.10 (0.27–0.77)	0.041^b^
RV IVCT (ms)	37.2 ± 4.1 (29.6–46.8)	36.9 ± 4.0 (30.2–47.1)	0.621^b^
RV IVRT (ms)	52.8 ± 5.6 (40.5–69.2)	50.9 ± 5.4 (41.1–68.4)	0.067^b^
RV ET (ms)	165.1 ± 12.2 (142.7–195.3)	167.4 ± 11.9 (145.6–198.1)	0.214^b^
TAPSE (mm)	7.71 ± 1.45 (4.7–11.2)	7.54 ± 1.29 (5.4–10.5)	0.404^b^
MAPSE (mm)	6.29 ± 1.12 (3.8–9.7)	6.25 ± 1.07 (4.0–8.6)	0.778^b^
Fetal heart rate (bpm)	145.59 ± 8.38 (124–176)	143.92 ± 9.27 (113–171)	0.180^b^
Left ventricular sphericity index (LVSI)	1.71 ± 0.19 (1.26–2.11)	1.70 ± 0.19 (1.31–2.26)	0.686^b^
Right ventricular sphericity index (RVSI)	1.69 ± 0.17 (1.32–2.10)	1.67 ± 0.20 (1.24–2.37)	0.551^b^
Cardiothoracic ratio (CTR)	0.29 ± 0.02 (0.24–0.35)	0.23 ± 0.04 (0.15–0.35)	< 0.001^a^
Amniotic fluid index (AFI, cm)	11.24 ± 1.41 (8.4–14.6)	17.92 ± 1.60 (13.4–21.5)	< 0.001^b^

Continuous variables are presented as mean ± standard deviation (range). *p* < 0.05 was considered statistically significant

*Abbreviations*: *MPI *Myocardial Performance Index, *IVCT *Isovolumetric Contraction Time, *IVRT *Isovolumetric Relaxation Time, *ET *Ejection Time, *TAPSE *Tricuspid Annular Plane Systolic Excursion, *MAPSE *Mitral Annular Plane Systolic Excursion, *LVSI *Left Ventricular Sphericity Index, *RVSI *Right Ventricular Sphericity Index, *CTR *Cardiothoracic Ratio, *AFI *Amniotic Fluid Index, *bpm* beats per minute

^a^ Mann–Whitney U test; ^b^ Student’s t-test

### Effect size and clinical significance

Cohen’s *d* analysis demonstrated clinically meaningful differences for several parameters (Table 4). The largest effect was observed for AFI (*d* = 1.35, very large), followed by CTR (*d* = 0.82, large) and LV MPI (*d* = 0.63, moderate–large). These findings support the presence of physiologic alterations in both fetal cardiac performance and fluid balance during maternal fasting.

**Table 4:** Effect size (Cohen’s *d*) of differences between fasting and control groups

Parameter	Mean (Fasting)	Mean (Control)	Cohen’s d	Interpretation
Middle Cerebral Artery PI	2.13	2.05	0.50	Medium effect
Left Ventricular MPI	0.54	0.49	0.63	Moderate–large effect
Right Ventricular MPI	0.56	0.53	0.41	Medium effect
Cardiothoracic Ratio (CTR)	0.29	0.23	0.82	Large effect
Amniotic Fluid Index (AFI)	11.24	17.92	1.35	Very large effect

Cohen’s *d* values quantify the standardized mean difference between fasting and control groups

According to Cohen’s convention: 0.2 = small, 0.5 = medium, and 0.8 or greater = large effect

*Abbreviations*: *PI* Pulsatility Index, *MPI *Myocardial Performance Index, *CTR *Cardiothoracic Ratio, *AFI *Amniotic Fluid Index

### Correlation analysis

Spearman’s correlation matrix revealed significant associations between cardiac and hemodynamic variables (Table 5). LV MPI correlated positively with RV MPI (*r* = 0.52, *p* < 0.001) and CTR (*r* = 0.33, *p* < 0.05), but negatively with AFI (*r* = − 0.42, *p* < 0.001). Similarly, MCA PI was inversely associated with AFI (*r* = − 0.36, *p* < 0.05). These results suggest that increased fetal cardiac load and cerebral vascular resistance are accompanied by reduced amniotic fluid volume, reflecting a potential systemic adaptation to maternal fasting.

**Table 5:** Spearman correlation matrix between fetal hemodynamic and functional parameters

Variable	LV MPI	RV MPI	CTR	AFI	MCA PI
LV MPI	—	0.52	0.33	**–0.42**	0.38
RV MPI	0.52	—	**0.39**	–0.35	0.29
CTR	0.33	**0.39**	—	–0.30	0.20
AFI	**–0.42**	–0.35	–0.30	—	**–0.36**
MCA PI	0.38	0.29	0.20	**–0.36**	—

Spearman’s rank correlation coefficients (*rₛ*) were calculated to assess associations between fetal cardiac function and hemodynamic parameters

Bold values denote significant correlations (*p* < 0.05)

*Abbreviations*: *LV MPI *Left Ventricular Myocardial Performance Index, *RV MPI *Right Ventricular Myocardial Performance Index, *CTR *Cardiothoracic Ratio, *AFI *Amniotic Fluid Index, *MCA PI *Middle Cerebral Artery Pulsatility Index

### Regression analysis

Multiple linear regression models identified fasting as an independent predictor of elevated LV MPI and reduced AFI after adjustment for gestational age (Table 6). Fasting status was significantly associated with higher LV MPI (β = 0.036, *p* = 0.002) and lower AFI (β = − 6.648, *p* < 0.001). Gestational age showed a modest positive association with LV MPI (β = 0.0078, *p* = 0.001) but had no significant effect on AFI (*p* = 0.291). The models explained 11% and 83% of the variance in LV MPI and AFI, respectively.

**Table 6:** Multiple linear regression analysis for predictors of fetal cardiac function (LV MPI) and amniotic fluid index (AFI)

Dependent Variable	Predictor	β Coefficient	SE	t	*p* Value	95% CI
LV MPI	Intercept	0.291	0.064	4.58	< 0.001	[0.166, 0.417]
	Fasting (vs. Control)	**0.036**	0.012	3.08	**0.002**	[0.013, 0.060]
	Gestational Weeks	**0.0078**	0.002	3.29	**0.001**	[0.003, 0.013]
AFI	Intercept	19.156	1.170	16.37	< 0.001	[16.849, 21.463]
	Fasting (vs. Control)	**–6.648**	0.217	–30.60	**< 0.001**	[–7.076, − 6.219]
	Gestational Weeks	–0.046	0.044	–1.06	0.291	[–0.133, 0.040]

Linear regression models were used to evaluate the independent effects of fasting status and gestational age on fetal cardiac function (LV MPI) and amniotic fluid volume (AFI)

R² = 0.11 for LV MPI model and R² = 0.83 for AFI model

Note. Values shown in bold indicate statistically significant predictors in the multivariable linear regression models (p < 0.05)

*Abbreviations*: *LV MPI *Left Ventricular Myocardial Performance Index, *AFI *Amniotic Fluid Index, *CI *Confidence Interval, *SE *Standard Error

### ROC curve analysis

**Fig. 1:**
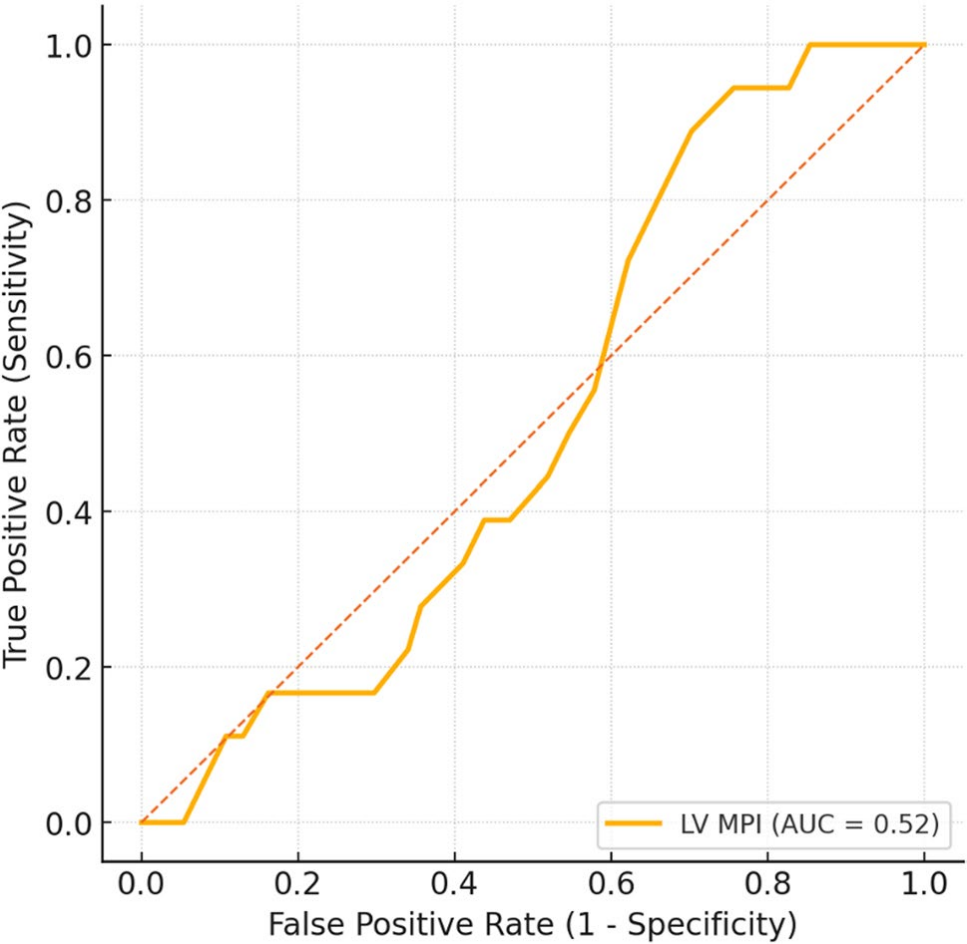
ROC Curve for LV MPI Predicting Low Amniotic Fluid (AFI < 10 cm

Receiver operating characteristic (ROC) analysis was performed to evaluate the ability of LV MPI to predict low amniotic fluid volume (AFI < 10 cm). The area under the curve (AUC) was 0.52 (95% CI: 0.46–0.58), indicating limited discriminative power of LV MPI in detecting pregnancies with reduced AFI (Table 7; Fig. 1). While a moderate correlation was observed between LV MPI and AFI, the low AUC indicates that LV MPI cannot be utilized as a reliable screening tool for reduced amniotic fluid in this cohort.

**Table 7:** ROC analysis for the predictive value of LV MPI in identifying low amniotic fluid (AFI < 10 cm)

Predictor	AUC (95% CI)	Optimal Cut-off	Sensitivity (%)	Specificity (%)	*p* Value
LV MPI	0.52 (0.46–0.58)	0.53	55	49	0.41

Receiver Operating Characteristic (ROC) analysis was conducted to determine the discriminative ability of LV MPI in predicting low amniotic fluid (AFI < 10 cm)

An AUC of 0.50 represents no discrimination, while values ≥ 0.70 indicate acceptable predictive accuracy

*Abbreviations*: *LV MPI *Left Ventricular Myocardial Performance Index, *AFI *Amniotic Fluid Index, *AUC *Area Under the Curve, *CI* Confidence Interval

## Discussion

This prospective cross-sectional study evaluated associations between maternal Ramadan fasting and fetal hemodynamic and myocardial parameters using comprehensive Doppler echocardiography.The results demonstrated significantly higher MCA PI, left and right ventricular myocardial performance indices (LV MPI, RV MPI), and CTR in fasting pregnancies, while the AFI was notably reduced. Conversely, umbilical and ductus venosus (DV) Doppler indices, as well as TAPSE, MAPSE, and heart rate, remained unchanged. These findings are associated with subclinical physiological cardiovascular changes rather than overt pathological compromise.

### Fetal cerebral hemodynamics

The increase in MCA PI observed in fasting pregnancies implies a mild rise in cerebrovascular resistance. Previous research has reported inconsistent effects of fasting on cerebral blood flow [5, 6]. Recent Doppler-based studies conducted during Ramadan have demonstrated largely preserved umbilical artery indices and perinatal outcomes, with only mild and transient variations in cerebral Doppler parameters and amniotic fluid volüme [12, 13].

While some authors found no significant differences in MCA or umbilical indices [5, 7], our results align with the physiological hypothesis that maternal hypoglycemia and dehydration toward the end of the fasting day may transiently increase cerebrovascular impedance [14, 15].

Cerebral autoregulation allows the fetus to maintain oxygen delivery despite short-term metabolic fluctuations [16]. Thus, elevated MCA PI in our cohort likely reflects metabolic vasoreactivity rather than hypoxic redistribution.

This adaptive response may differ depending on the duration of fasting, gestational age, and climatic conditions, particularly during longer summer fasts [4].

In our study, although the ductus venosus pulsatility index did not differ significantly between groups, a borderline trend toward higher values was observed in the fasting group (*p* = 0.054). While this finding does not meet the threshold for statistical significance, it may indicate subtle alterations in venous return or fetal preload conditions in response to maternal fasting. Importantly, the absence of abnormal ductus venosus waveform morphology, particularly the preservation of the a-wave, suggests that these changes are unlikely to represent clinically overt cardiac compromise. Rather, they appear to reflect a subclinical component of the fetal cardiovascular adaptive response, consistent with the preserved systolic function observed as assessed by TAPSE and MAPSE.

### Fetal myocardial performance

The increase in LV and RV MPI among fasting women represents the most notable and novel finding of this study. MPI integrates both systolic and diastolic performance by combining isovolumetric contraction and relaxation times relative to ejection time [11]. Recent systematic reviews emphasize that the myocardial performance index is a sensitive marker of subclinical fetal cardiac adaptation and may detect functional changes even when conventional Doppler parameters remain within normal limits [17].

Previous studies have shown that elevated MPI is an early sign of ventricular loading alteration in conditions such as intrauterine growth restriction and gestational diabetes [9, 10, 18].

The observed MPI elevation in fasting pregnancies, independent of gestational age, may result from subtle reductions in preload or changes in myocardial relaxation secondary to transient maternal dehydration and reduced glucose supply [14, 19]. In addition to reduced preload related to transient maternal dehydration, a potential contribution of increased afterload should also be considered when interpreting the observed elevation in MPI. Although umbilical artery pulsatility index remained unchanged, suggesting preserved placental vascular resistance, the concomitant increase in middle cerebral artery PI may reflect subtle fetal circulatory adjustments in response to altered maternal metabolic conditions. Such adaptations could transiently influence ventricular loading conditions without manifesting as overt placental Doppler abnormalities. Therefore, the increased MPI observed in fasting pregnancies likely represents a combined effect of mild preload reduction and subtle afterload-related circulatory modulation rather than isolated impairment of myocardial performance.

Importantly, TAPSE and MAPSE values remained stable between groups, indicating preserved systolic contractility and supporting the concept that diastolic parameters may be more sensitive to metabolic shifts as an early myocardial response [9]. Although absolute TAPSE and MAPSE values may appear lower when compared with reference ranges derived from late gestation, both fetal TAPSE and MAPSE are strongly dependent on gestational age. Given the mean gestational age of approximately 27 weeks in our cohort, the observed TAPSE and MAPSE values fall within the expected range for this gestational window [20].

### Cardiac morphology and circulatory load

An increased cardiothoracic ratio (CTR) was also observed among fasting women. Although CTR has been associated with pathological cardiomegaly in fetal compromise [21], modest increases in CTR without changes in sphericity indices (LVSI, RVSI) likely indicate temporary volume adaptation or mild preload elevation.

Comparable findings have been reported in adaptive fetal remodeling under mild hemodynamic stress [22, 23]. The absence of structural deformation or venous congestion in our study suggests that these cardiac modifications are functional and likely transient rather than overtly pathologic.

### Amniotic fluid and placental perfusion

The reduction in AFI observed among fasting women is consistent with several previous studies [24, 25], which attributed the decrease primarily to maternal dehydration and reduced renal plasma flow.

However, other investigations did not find significant AFI changes [5, 7], likely due to differences in fasting duration, environmental conditions, and fluid intake habits [4, 26].

In our cohort, conducted in southeastern Türkiye during long fasting hours, lower AFI likely reflects maternal water restriction rather than placental dysfunction.

The negative correlation between AFI and MPI further suggests an interrelated compensatory mechanism: as placental flow and amniotic fluid decrease, fetal myocardial workload subtly increases to maintain adequate circulation [9, 10].

### Integrative physiological interpretation

Collectively, the combination of increased MPI, elevated MCA PI, and reduced AFI represents a coordinated, subclinical fetal response to transient maternal metabolic and hydration changes.

This adaptive pattern supports the concept of fetal cardiovascular resilience, wherein the fetus maintains hemodynamic homeostasis through autonomic and myocardial adjustments [11, 16].

Such physiological buffering likely explains why large population studies found no adverse effects of Ramadan fasting on birth weight or neonatal outcomes [8, 27].

Nevertheless, this adaptive reserve may be limited in pregnancies with compromised placental function, such as preeclampsia or fetal growth restriction [28–30].

### Clinical implications

Our results reinforce that Ramadan fasting is generally safe in healthy pregnancies, provided that hydration and caloric intake are adequate [1, 2].

However, the observed Doppler-based changes highlight the need for individualized counseling and monitoring.

Clinicians should recommend adequate fluid intake during non-fasting hours, shorter fasting durations when possible, and mid-Ramadan fetal Doppler assessment for women at increased risk.

In cases with pre-existing maternal or placental compromise, temporary fasting exemptions should be considered to prevent potential fetal stress [5, 6].

#### Strengths and Limitations

The strengths of this study include its prospective design, relatively homogeneous cohort, and comprehensive fetal cardiac evaluation using multiple Doppler-derived functional and hemodynamic indices. The application of advanced statistical approaches, including effect size analysis, multivariable regression, and ROC curve evaluation, enhanced the robustness and interpretative depth of the findings. However, several limitations should be acknowledged. First, the cross-sectional design does not allow causal inference, and fetal echocardiography was not performed longitudinally before and after Ramadan within the same individuals; therefore, the temporal course and eversibility of the observed Doppler changes could not be directly assessed. Second, maternal biochemical and metabolic markerssuch as glucose, insulin, ketone bodies, or objective hydration parameters) were not measuredconcurrently, limiting mechanistic interpretation of fetal cardiovascular adaptations. Third, long-term neonatal outcomes were not evaluated, and thus the persistence and clinicalsignificance of these subclinical findings beyond the antenatal period remain uncertain.Finally, as this was a single-center study conducted under specific seasonal and geographic conditions, the generalizability of the results may be limited, underscoring the need for multicenter studies in diverse environmental settings. 

### Future directions

Future studies should integrate maternal metabolic profiling, serial fetal Doppler monitoring, and postnatal echocardiography to delineate the temporal dynamics of these adaptations.

Emerging technologies, including fetal strain imaging, 3D cardiac volumetry, and AI-based Doppler waveform analysis, may provide greater insight into subtle myocardial and vascular changes.

Expanding research to include high-risk pregnancies will be critical to identify thresholds where fasting transitions from adaptive to detrimental.

## Conclusion

In conclusion, maternal Ramadan fasting was associated with mild and potentially adaptive fetal cardiovascular changes, characterized by increased myocardial performance index and middle cerebral artery pulsatility index, along with reduced amniotic fluid volume, without overt Doppler evidence of fetal compromise.

These findings suggest that the fetal cardiovascular system demonstrates a degree of physiological adaptability to short-term maternal metabolic and hydration changes during fasting. However, given the cross-sectional nature of the study, causality and reversibility cannot be established, and careful individualized assessment remains essential. Further longitudinal studies are warranted to clarify the persistence and long-term clinical significance of these subtle hemodynamic alterations.

## Supplementary Information

Supplementary Material 1.

Supplementary Material 2.

## Data Availability

The datasets generated and analyzed during the current study are available from the corresponding author, Dr. Deniz Taşdemir (email: tasdemir_deniz1982@hotmail.com), upon reasonable request. All de-identified data are stored securely in the Şanlıurfa Training and Research Hospital database.
