# Supplementary material for: Maternal Ramadan fasting and fetal cardiac function: subclinical hemodynamic alterations revealed by doppler evaluation
Source: BMC Pregnancy Childbirth. 2026 Feb 5;26:235. doi: 10.1186/s12884-026-08683-4 (PMC12964637; doi:10.1186/s12884-026-08683-4)
Supplement: Supplementary file 1 — Supplementary Material 1. [file 12884_2026_8683_MOESM1_ESM.docx]

# CONSORT Checklist (Adapted “Where Applicable” for the Revised Manuscript)

Manuscript title: Maternal Ramadan Fasting and Fetal Cardiac Function: Subclinical Hemodynamic Alterations Revealed by Doppler Evaluation

Author: Dr. Deniz Taşdemir

Journal: BMC Pregnancy and Childbirth

Trial registration: ClinicalTrials.gov Identifier: NCT06900257

Study type: Prospective observational study evaluating effects of maternal fasting (no randomization)

Statement: This study adheres to the CONSORT recommendations where applicable for reporting prospective interventional/observational studies.

## Title and Abstract

| Item No | Checklist item | Reported on page |
| --- | --- | --- |
| 1a | Identification as a randomized trial in the title | Not applicable (observational prospective study) |
| 1b | Structured summary of trial design, methods, results, and conclusions | Abstract, pp. 1-3 |

## Introduction

| Item No | Checklist item | Reported on page |
| --- | --- | --- |
| 2a | Scientific background and rationale for the trial | Introduction, pp. 3-4 |
| 2b | Specific objectives or hypotheses | End of Introduction, p. 4 |

## Methods

| Item No | Checklist item | Reported on page |
| --- | --- | --- |
| 3a | Description of trial design | Methods, pp. 4-7 (prospective two-group observational comparison) |
| 4a | Eligibility criteria for participants | Methods, Study Population, pp. 4–5 |
| 5 | Interventions for each group | Methods, pp. 4–5 |
| 6a | Primary and secondary outcome measures | Methods, Ultrasound and Doppler Protocol, pp. 6–7 |
| 7a | How sample size was determined | Methods, Power and Sensitivity Analysis, p. 8 |
| 8–10 | Randomization details | Not applicable (non-randomized study) |
| 11a | Blinding | Single operator, not group-blinded |
| 12a | Statistical methods | Methods, Statistical Analysis, pp. 7-8 |

## Results

| Item No | Checklist item | Reported on page |
| --- | --- | --- |
| 13a | Participant flow | Methods, p. 4 |
| 14a | Recruitment dates | Methods, p. 4 |
| 15 | Baseline data | Table 1 |
| 16 | Number analyzed in each group | Results, p. 10 |
| 17a | Outcome results and effect size | Results, Tables 2–7, pp. 8-9 |
| 18 | Subgroup or adjusted analyses | Regression, correlation, ROC, p. 10 |
| 19 | Harms or unintended effects | None observed |

## Discussion

| Item No | Checklist item | Reported on page |
| --- | --- | --- |
| 20 | Trial limitations | Strengths and Limitations, pp. 14-15 |
| 21 | Generalizability | Clinical Implications, p. 14 |
| 22 | Interpretation considering benefits/harms | Discussion and Conclusion, pp. 11-16 |

## Other Information

| Item No | Checklist item | Reported on page |
| --- | --- | --- |
| 23 | Registration number and name of registry | NCT06900257 (ClinicalTrials.gov) p.16 |
| 24 | Access to full protocol | Available at ClinicalTrials.gov and Harran University Ethics Committee p. 16 |
| 25 | Sources of funding and role of funders | No funding was received for this study. p. 17. |
| — | CONSORT Statement added under Declarations section | Declaration. p. 16. |

This study was conducted in accordance with the CONSORT Statement where applicable. Although the design was a prospective, non-randomized observational comparison, all relevant items concerning participant flow, outcomes, and analysis were reported following CONSORT principles.

# File Description for Submission

File name: Updated_CONSORT_Checklist_Deniz_Tasdemir.docx

File description (for BMC submission portal):
CONSORT 2010 Checklist (applicable items only).
This checklist was completed in accordance with CONSORT guidelines where applicable. Although the study was designed as a prospective observational comparison rather than a randomized trial, all relevant items concerning study design, participant flow, outcomes, and statistical analysis were reported following CONSORT principles.
