# Supplementary figures and images for: Maternal Ramadan fasting and fetal cardiac function: subclinical hemodynamic alterations revealed by doppler evaluation
Source: BMC Pregnancy Childbirth. 2026 Feb 5;26:235. doi: 10.1186/s12884-026-08683-4 (PMC12964637; doi:10.1186/s12884-026-08683-4)

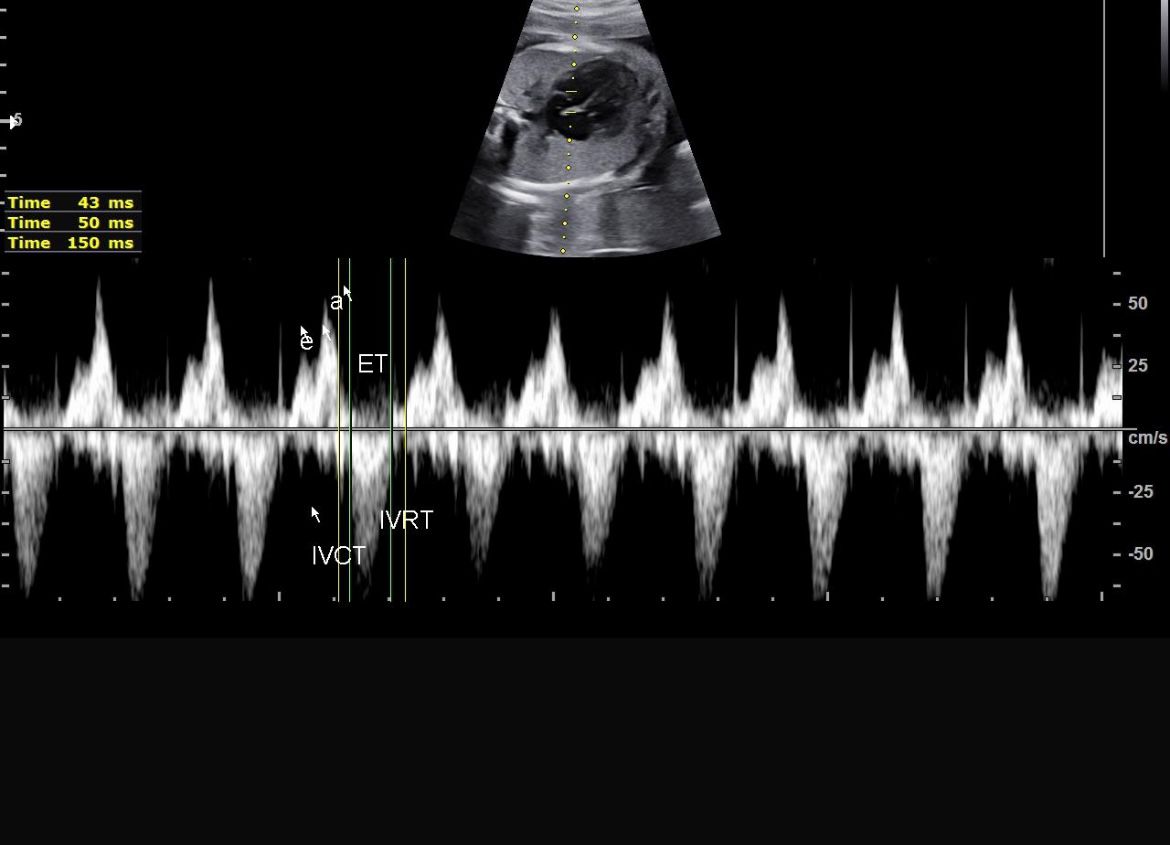

Supplement: Supplementary file 2 — Supplementary Material 2. [file 12884_2026_8683_MOESM2_ESM.jpg]
